# Loss of KIBRA function activates EGFR signaling by inducing AREG

**DOI:** 10.18632/oncotarget.25724

**Published:** 2018-07-06

**Authors:** Ashley L. Mussell, Kayla E. Denson, He Shen, Yanmin Chen, Nuo Yang, Costa Frangou, Jianmin Zhang

**Affiliations:** ^1^ Department of Cancer Genetics & Genomics, Roswell Park Cancer Institute, Buffalo, NY, USA; ^2^ Department of Anesthesiology, Jacobs School of Medicine & Biomedical Sciences, University at Buffalo, The State University of New York, Buffalo, NY, USA; ^3^ Harvard T.H. Chan School of Public Health, Molecular and Integrative Physiological Sciences, Boston, MA, USA; ^4^ Current address: Frontier Science Foundation, Amherst, NY, USA

**Keywords:** Hippo pathway, EGFR signaling, 3D culture, breast tumorigenesis

## Abstract

The Hippo signaling pathway is a central regulator of organ size, tissue homeostasis, and tumorigenesis. KIBRA is a member of the WW domain-containing protein family and has recently been reported to be an upstream protein in the Hippo signaling pathway. However, the clinical significance of KIBRA deregulation and the underlying mechanisms by which KIBRA regulates breast cancer (BC) initiation and progression remain poorly understood. Here, we report that KIBRA knockdown in mammary epithelial cells induced epithelial-to-mesenchymal transition (EMT) and increased cell migration and tumorigenic potential. Mechanistically, we observed that inhibiting KIBRA induced growth factor-independent cell proliferation in 2D and 3D culture due to the secretion of amphiregulin (AREG), an epidermal growth factor receptor (EGFR) ligand. Also, we show that AREG activation in KIBRA-knockdown cells depended on the transcriptional coactivator YAP1. Significantly, decreased expression of KIBRA is correlated with recurrence and reduced BC patient survival. In summary, this study elucidates the molecular events that underpin the role of KIBRA in BC. As a result, our work provides biological insight into the role of KIBRA as a critical regulator of YAP1-mediated oncogenic growth, and may have clinical potential for facilitating patient stratification and identifying novel therapeutic approaches for BC patients.

## INTRODUCTION

Breast cancer (BC) is the second leading cause of death in women in the United States and is the most common cancer that affects women. Knowledge of the underlying mechanisms of BC progression and metastasis has increased considerably throughout the past 10 years. Pathways and protein interactions that drive this disease are continually being identified and characterized; however, the key signaling nodes remain elusive [1].

The Hippo signaling pathway was first identified in *Drosophila melanogaster* and controls cell proliferation, apoptosis, and organ size [2, 3]. This pathway is necessary for normal tissue growth and organ size control, and disruptions/aberrations in Hippo signaling are involved in tumorigenesis [4, 5]. In breast cancer, deregulation of Hippo signaling can drive progression through the activation of its effector molecules YAP1 (Yes-associated protein 1) and TAZ (transcriptional co-activator with PDZ binding motif) [4]. The nuclear translocation of YAP1 and TAZ is associated with increased breast cancer progression, metastasis, epithelial-to-mesenchymal transition (EMT), epithelial stem cell regeneration, and therapeutic resistance [6]. The tight regulation of this pathway ensures the precise control of cell cycle progression and apoptosis. Upon activation by various external stimuli, Hippo signaling induces a serine-threonine kinase cascade [7]. In mammals, MST1/2 (mammalian Ste20-like serine/threonine kinase 1/2) is phosphorylated, as are LATS1/2 (large tumor-suppressor kinase 1/2) upon interaction with SAV1 [3]. In combination with non-receptor tyrosine phosphatase (PTPN14) and KIBRA, LATS1/2 phosphorylate the key effectors of Hippo signaling, YAP1 and TAZ [8, 9]. Phosphorylated YAP1/TAZ interact with the 14-3-3 proteins, sequestering YAP1/TAZ in the cytoplasm [10–12]. β-TrCP is recruited to phosphorylated YAP/TAZ, which prompts their ubiquitination and subsequent proteasome degradation [13].

In humans, impaired Hippo signaling has been reported for several types of cancer. In line with this observation, components of the Hippo pathway are frequent targets of aberrant gene regulation and epigenetic silencing in BC [4]. Nonetheless, while the oncogenic functions of YAP1/TAZ are well established, many upstream regulators of this signaling cascade remain elusive. KIBRA (also known as WWC1) was initially discovered in 2003 in a yeast two-hybrid screen and was found to contain two amino-terminal WW domains: an internal C2-like domain and a carboxy-terminal acid-rich stretch [14]. The WW domain of KIBRA recognizes the PPxY motif (P, proline; Y, tyrosine; and x, any amino acid) and mediates interactions with proteins containing this motif [15]. KIBRA has been shown to exert its tumor suppressive effects through interaction with Merlin and Expanded, two upstream regulators of Hippo signaling [16–18]. LATS1 was recently shown to be activated independently by KIBRA and PTPN14 and cooperatively by the KIBRA/PTPN14 complex [8]. Notwithstanding these findings, little is known about the detailed molecular mechanisms linking KIBRA to the regulation of the Hippo pathway and tumorigenesis. Thus, the mechanisms of the tumor suppressor function of KIBRA need to be understood from a therapeutic standpoint and may provide additional insights into treatment.

In this study, we reveal that the loss of function of KIBRA in MCF10A mammary epithelial cells induced EMT, anchorage-independent growth and growth factor-independent cell proliferation in 2D and 3D culture. Mechanistically, the downregulation of KIBRA induced epidermal growth factor receptor (EGFR) activation by promoting the expression of the EGFR ligand amphiregulin (AREG). Furthermore, we demonstrated that AREG activation by KIBRA loss of function is dependent on YAP1.

## RESULTS

### Loss of function of KIBRA induces EMT and mammary epithelial cell transformation

**Figure 1:**
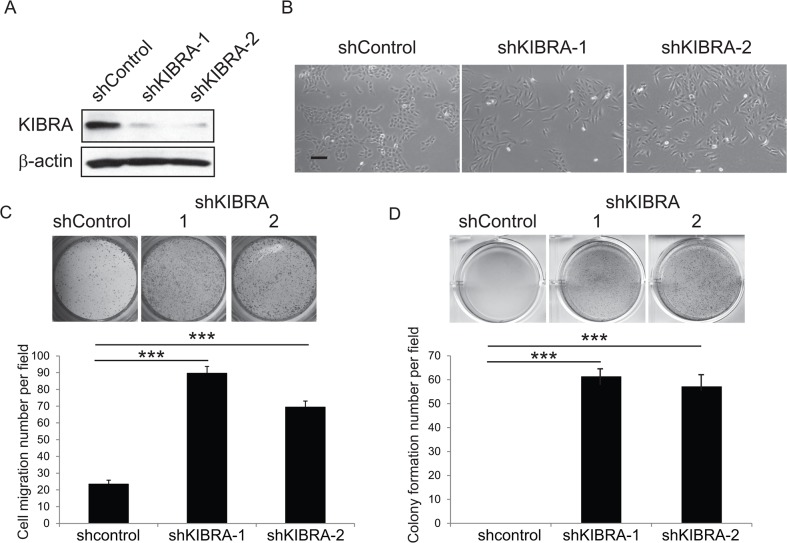
Loss of KIBRA function induces EMT and mammary epithelial cell transformation **(A)** Immunoblot demonstrating the efficient knockdown of KIBRA using two individual shRNAs compared to shControl. β-actin was used as the loading control. **(B)** Representative cell morphological images of shControl and shKIBRA MCF10A cells. **(C)** Representative images and quantification of Boyden chamber cell migration assay for shControl or shKIBRA cells. **(D)** Representative images and quantification of the colony formation in soft agar assay for shControl or shKIBRA cells.

We previously demonstrated that KIBRA interacts with PTPN14 and synergistically activates LATS1 and inhibits YAP1 oncogenic function [8]. To further understand the functional role of KIBRA in breast cancer, we knocked down KIBRA in MCF10A mammary epithelial cells using RNAi. The transduction of two independent shRNA constructs into MCF10A cells led to a significant reduction in KIBRA expression (Figure 1A). Consistent with a previous report [19], we observed that KIBRA knockdown caused a transition from an epithelial-like cell morphology to a mesenchymal-like cell morphology (Figure 1B); this finding indicated that the loss of KIBRA function induced EMT. Correspondingly, we detected increased migration (Figure 1C) and soft agar colony formation (Figure 1D) of KIBRA-knockdown cells *in vitro*. Together, these data suggest that the loss of KIBRA causes mammary epithelial cell transformation.

### KIBRA knockdown induces growth factor-independent cell proliferation

**Figure 2:**
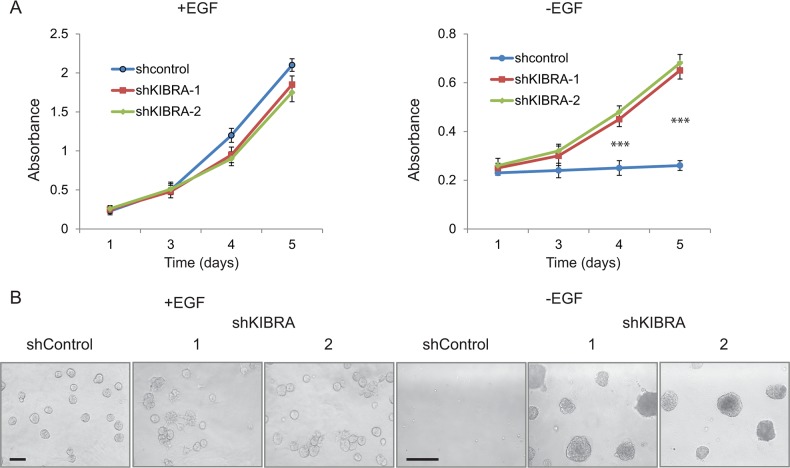
KIBRA knockdown induces growth factor-independent cell proliferation **(A)** shControl and shKIBRA 2D culture cell proliferation in the presence or absence of EGF was detected by MTT assay. (p<0.001^***^). **(B)** shControl and shKIBRA 3D acini formation assays were performed in the presence or absence of EGF.

The activation of mitogenic growth signals through the secretion of growth factors or the activation of growth factor receptors is a key hallmark of tumor cells [20]. MCF10A cells are immortalized, non-transformed human mammary epithelial cells that are dependent on growth factors for proliferation and survival [21]. To determine whether KIBRA knockdown affects cell proliferation in the presence or absence of epidermal growth factor (EGF), we performed MTT assays in 2D culture. As shown in Figure 2A, we did not detect a notable change in shKIBRA cell proliferation in the presence of EGF, but we did detect a marked difference between these cells in the absence of EGF (Figure 2A). In 3D culture, compared to the control, KIBRA knockdown promoted multi-acini formation in the presence of EGF (Figure 2B). More interestingly, KIBRA knockdown induced acini formation in the absence of EGF (Figure 2B). Together, these results suggest that the loss of KIBRA function may activate mitogenic growth signals through the activation of growth factor receptors or the secretion of growth factor(s).

### KIBRA knockdown induces the secretion of AREG, an EGFR ligand

**Figure 3:**
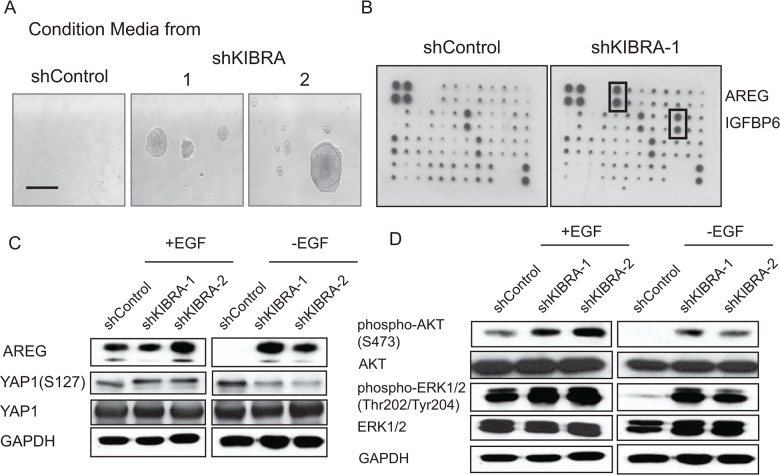
KIBRA knockdown induces the secretion of the EGFR ligand AREG **(A)** Conditioned media collected from shControl or shKIBRA 3D cell cultures was used to treat parental MCF10A cells. **(B)** Human growth factor/cytokine antibody array analyses were performed using conditioned media from shControl- or shKIBRA-transduced cells grown in the absence of EGF. Four positive and four negative controls are shown in the upper left corner. **(C)** Immunoblot detection of AREG, phosphor-YAP1 (S127) and YAP1 expression in the presence or absence of EGF in shControl or shKIBRA cells. GAPDH was used as the loading control. **(D)** AKT and ERK activation was detected in the presence or absence of EGF in shControl or shKIBRA cells by immunoblot. GAPDH was used as the loading control.

To determine whether KIBRA knockdown leads to the secretion of growth factor(s) or the activation of growth factor receptors, we performed a conditioned media experiment (Figure 3A). Conditioned media was harvested from either shGFP or two shKIBRA 3D cell cultures in the absence of growth factor; the media was then applied to parental MCF10A cells. Interestingly, the conditioned media collected from shKIBRA cells promoted parental MCF10A cell growth. In contrast, the conditioned media from shGFP cells did not increase proliferation (Figure 3A).

Next, to identify the potential growth factor(s) released from shKIBRA cells, we performed growth factor/cytokine array analyses using conditioned media collected from shGFP or shKIBRA cells. We found that AREG and IGFBP6 were highly enriched in the conditioned media from shKIBRA cells compared to that from shGFP cells (Figure 3B). Furthermore, we confirmed AREG upregulation in shKIBRA cells by immunoblot (Figure 3C). We did not observe IGFBP6 upregulation in shKIBRA cells (data not shown). Consistent with our previously finding that IGFBP6 is a secreted growth factor from MCF10A cells when the cells undergo proliferation [22]. These results indicate that KIBRA knockdown induces the secretion of AREG, a growth factor and EGFR ligand.

### AREG upregulation in shKIBRA cells depends on YAP1 expression

AREG is a known ligand of EGFR and can drive downstream signaling pathways [23]. To determine whether PI3K-AKT and MAPK-ERK signaling are activated in KIBRA-knockdown cells, we detected AKT and ERK activation by immunoblot. We observed an increase in p-AKT and p-ERK levels in shKIBRA cells in the absence of EGF; these results indicated the activation of PI3K-AKT and MAPK-ERK signaling in KIBRA-knockdown cells (Figure 3D).

**Figure 4:**
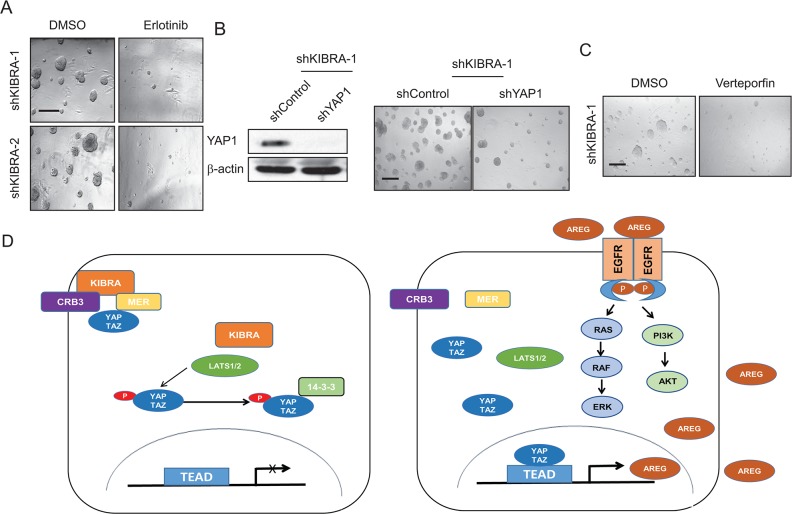
AREG upregulation in shKIBRA cells depends on YAP1 expression **(A)** shKIBRA 3D acini formation assays were performed with erlotinib (10 μM) treatment in the absence of EGF. **(B)** Immunoblot demonstrating the efficient knockdown of YAP1 in shKIBRA cells (left panel). β-actin was used as the loading control. shYAP1/shKIBRA 3D acini formation assays were performed in the absence of EGF (right panel). **(C)** Treating KIBRA-knockdown cells in the 3D acini formation assay with verteporfin (1 μM) abolished acini formation. **(D)** The presence of KIBRA induces canonical Hippo signaling to restrict YAP1/TAZ to the cytoplasm. However, in the absence of KIBRA, YAP1/TAZ can be translocated into the nucleus, where they drive AREG expression, thus leading to EGFR activation and EGF-independent cell proliferation.

To confirm that KIBRA knockdown induces growth factor-independent cell proliferation through EGFR activation, we treated shKIBRA 3D cultures with the EGFR inhibitor erlotinib. We found that erlotinib treatment completely inhibited 3D acini formation in shKIBRA cells (Figure 4A). We previously reported that YAP1 binds directly to the AREG promoter and activates AREG expression [22]. Accordingly, to determine whether KIBRA knockdown induces AREG secretion in a YAP1 expression-dependent manner, we knocked down YAP1 in shKIBRA MCF10A cells (Figure 4B). YAP1 knockdown dramatically reduced acini formation in shKIBRA cells in the absence of EGF (Figure 4B). Furthermore, treatment with the YAP1 inhibitor Verteporfin (VP) completely blocked growth factor-independent acini formation in shKIBRA cells (Figure 4C). Taken together, our data demonstrate that the loss of KIBRA function induces growth factor-independent cell proliferation via a mechanism dependent on YAP1 activation (Figure 4D).

### KIBRA gene expression level correlates with clinicopathological features of BC patients

**Figure 5:**
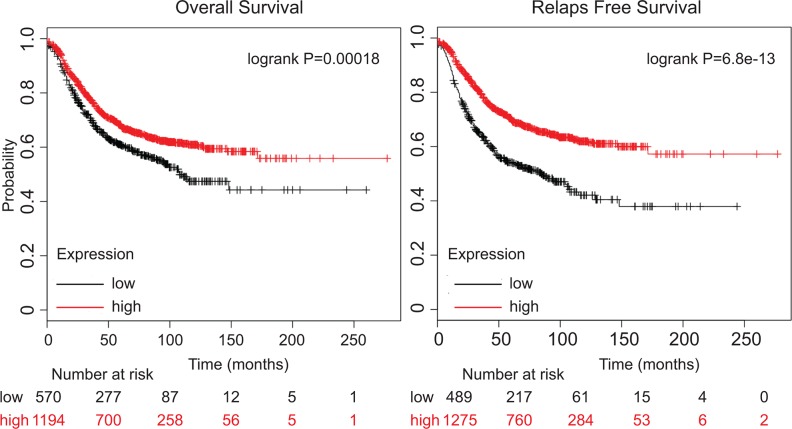
Low KIBRA expression predicts poor breast cancer patient outcome Kaplan-Meier overall survival (OS) and relapse-free survival (RFS)analysis of breast cancer patients using a median split of KIBRA gene expression (KM-plotter). The Log-Rank test was used to measure the statistical difference between the high and low KIBRA groups for Kaplan-Meier curves.

Finally, to investigate whether our findings have clinical relevance, the correlation between the relative mRNA expression levels of KIBRA, and important clinicopathological features were examined by univariate Kaplan-Meier analyses (Methods). A positive association between decreased KIBRA expression and both reduced median overall survival (OS) and reduced relapse-free survival (RFS) within five years of diagnosis was observed using several independent patient datasets (Figure 5). In agreement with these findings, a separate multivariate analysis of prognostic factors with a Cox proportional-hazards model confirmed that low KIBRA expression was a robust predictor of poor survival in BC and remained significant when adjusting for other prognostic factors, such as histological type and tumor grade.

## DISCUSSION

KIBRA is a signal transduction protein that was initially determined to be expressed mainly in the kidney and brain [24]. Genetic screens in Drosophila revealed that KIBRA forms a complex with Merlin and Expanded, the upstream components of the Hippo signaling pathway, and thus phosphorylates Yorkie (Yki) to inhibit its activity [16–18]. In mammalian cells, KIBRA interacts directly with LATS1/2 and is independent of MST1/2 [9]. We previously reported that KIBRA and PTPN14 form a complex that regulates LATS1 function [8]. In addition, it has been recently reported that the apical-basal polarity protein CRB3 interacts and stabilize KIBRA in mammary epithelial cells [25]. The mammalian KIBRA/WWC1 protein contains another two similar proteins (WWC2 and WWC3) [26]. It has been reported that the WWC genes are differentially regulated in a tissue-specific manner [27]. Similar to KIBRA, both WWC2 and WWC3 capable activate LATS1/2 and negatively regulate YAP1 oncogenic functions [27–30].

KIBRA methylation has been observed in B-cell acute lymphocytic leukemia (B-ALL), chronic lymphocytic leukemia (CLL), gastric cancer (GC) and clear cell renal cell carcinomas (ccRCC) [31–34]. In conjunction with the stem cell transcription factor Sox2, KIBRA plays an important role in maintaining cancer stem cell (CSC) properties and tumorigenicity in osteosarcomas [35]. Down-regulation of WWC2 has been reported to associated with advanced hepatocellular carcinomas (HCCs) [36]. In addition, decreased expression of WWC3 also has been found correlated with poor prognosis of GC [37].

In breast cancer, it has been reported that reduced KIBRA expression correlates with the claudin-low subtype of breast cancer [19]. Using a transgenic mouse model, Knight et al recently identified KIBRA as a major contributor to the effects of 5q loss on breast tumor growth and metastatic progression [38]. Interestingly, we also found 21 KIBRA mutations in breast cancer patients in The Cancer Genome Atlas (TCGA) dataset (Supplementary Figure 1 and Supplementary Table 1). The majority of these KIBRA mutations are missense mutations, and further characterization of these KIBRA mutations in breast cancer will provide better insight into KIBRA deregulation in breast cancer.

In this study, we demonstrate that losing KIBRA function activates EGFR signaling through the YAP1-dependent activation of AREG. Furthermore, we show that decreased KIBRA expression correlates with reduced BC patients’ OS and RFS. Notably, during the period our manuscript was under review, it was reported that luminal BC patients with endocrine therapy and KIBRA-low expression had an RFS disadvantage over those who were positive for KIBRA [39]. Accordingly, the correlation between expression of WWC2/WWC3 and clinical features of breast cancer must be further determined. Nonetheless, using genetic testing, it may be possible to assess the malignant progression of breast cancer based on KIBRA expression levels. Moreover, this approach might enable early intervention in breast cancer patients and provide novel therapeutic avenues for breast cancer treatment.

## MATERIALS AND METHODS

### Tissue culture

MCF10A cell was previously received from Dr. Joan Brugge's lab [21] and has been recently authenticated by STR profiling and test for mycoplasma contamination. The cell culture was performed as described previously [40, 41]. Briefly, MCF10A cells were grown in DMEM/F12 medium (Corning CellGro; NY) supplemented with 5% horse serum (Invitrogen; MA), 20 ng/ml EGF (ProSpec; NJ), 0.5 μg/mg hydrocortisone, 100 ng/ml cholera toxin, and 10 μg/ml insulin (Sigma; MO). For EGF-independent cell growth experiments, cells were plated and maintained in growth media for 24 h before being washed with PBS and incubated in assay media (complete MCF10A growth media without EGF). All cells were cultured in a humidified atmosphere of 95% air and 5% CO_2_ at 37°C.

### shRNA constructs

shRNA hairpins targeting human KIBRA sequences were obtained from the RNAi Consortium (The Broad Institute; MA). The target sequences used are listed in the 5′-3′ direction: shControl: CAACAAGATGAAGAGCACCAA; shKIBRA-1: TCAGATTGCGCCTTCGATATG; and shKIBRA-2: CCTTCACCAGAAGACCTTAAG.

shKIBRA constructs were generated in the pLKO.1 vector at the AgeI/EcoRI sites. Lentiviral packaging and the transient transfection of 293T cells were performed as described previously [42].

### Cell migration assay

Transwell cell migration assays were performed as described previously [42].

### Colony formation (soft agar assay)

Soft agar assays were performed as previously described [42]. Briefly, 2.0 mL of 0.5% agarose (Sigma-Aldrich; MO) was plated in 6-well plates as the base layer; 50,000 shControl or shKIBRA cells were suspended in 1.5 mL of 0.4% agarose and were plated on top of the base layer. Anchorage-independent growth was assessed by counting the colonies after two weeks of growth. All soft agar assays were repeated in at least three separate experiments.

### 3D acini formation assay

MCF10A 3D acini formation assays were performed as previously described [40, 41]. Briefly, 4.0×10^3^ shControl-transduced (control) or shKIBRA lentiviral-transduced MCF10A cells after puromycin selection for 72 hours were plated in 3D culture chambers and cultured with assay media in 5% growth factor-reduced Matrigel (#354230; Corning; MA) in presence of 5ng/ml EGF or absent of EGF. Assay media was replaced every 4 days. The assays were repeated in 3 independent experiments. Verteporfin was purchased from Fisher Scientific (53-051-0), and erlotinib was purchased from LC Laboratories (E-4007).

### Western blotting and antibodies

Cell lysates were created using RIPA buffer (Boston Bio-Products; MA) supplemented with protease and phosphatase inhibitors (Thermo Scientific; MA). Briefly, the sample proteins (30 or 40 μg) were separated by SDS-PAGE and then transferred onto PVDF membranes (EMD Millipore; MA). After blocking with 5% BSA or non-fat milk for 1 h, the membranes were incubated with primary antibodies overnight at 4°C. The next day, the membranes were incubated with an anti-rabbit or mouse secondary antibody (Bio-Rad; CA) for 1 h. Finally, the proteins were detected using ECL Plus Western Blotting Detection Reagents (GE Healthcare; PA). The cytokine/growth factor array analysis kit (AAH-GF-1) was purchased from RayBiotech The following primary antibodies were used for immunoblot: anti-KIBRA (# 8774), anti-phospho-YAP1 (S127) (#13008), anti-AKT (# 4685), anti-phospho-AKT (# 4060), anti-ERK (# 4695) and anti-phospho-ERK (# 9101) from Cell Signaling Technology, MA; anti-AREG (16036-1-AP) from Proteintech, IL; anti-YAP1 (SC-15407) from Santa Cruz Biotechnology, CA; anti-GAPDH (Y1041) and β-actin (Y1051) from Ubiquitin-Proteasome Biotechnologies, CO.

### Conditioned media assays

Cells were plated in 3D culture chambers as described previously [41]. Every 4 days, fresh conditioned media from shControl or shKIBRA cells was harvested and applied to normal MCF10A cells. Images were taken 20 days after the first treatment with conditioned media. The assays were repeated in 3 independent experiments.

### Patient survival analysis (Kaplan-Meier estimate)

Correlation between clinicopathologic factors and KIBRA gene expression score was tested using the chi-square test with the exact method using Monte Carlo estimation. Kaplan-Meier curves were created for both overall survival (OS), and relapse-free survival (RFS), and log-rank tests were used to compare KIBRA gene expression stratified by antibody status [estrogen receptor (ER) or progesterone receptor (PR) positive), and HER2 positive. Next, to analyze the prognostic value KIBRA, the patient cohorts were divided into two groups according to the median (or upper/lower quartile) expression of KIBRA. Multivariable survival models were fit using Cox proportional hazards model. Final models were chosen using backward selection, with a removal alpha of 0.05 using the following datasets were downloaded from NCBI GEO: GSE11121, GSE12093, GSE12276, GSE1456, GSE16391, GSE16446, GSE16716, GSE17705, GSE17907, GSE19615, GSE20271, GSE2034, GSE20685, GSE20711, GSE21653, GSE2603, GSE26971, GSE2990, GSE31519, GSE3494, GSE37946, GSE42568, GSE45255, GSE4611, GSE4922, GSE5327, GSE6532, GSE7390 and GSE9195. All p-values were two-sided unless otherwise stated and considered statistically significant at the 0.05 level. The final multivariate survival model incorporated age, pathologic stage, and ER status based on these criteria. All statistical analyses were performed using SAS (version 9.4; SAS Institute; Cary, NC, USA).

### Statistical analysis

All data are representative of three independent experiments. P-values were determined using two-tailed Student's *t*-tests (p<0.05^*^, p<0.01^**^, p<0.001^***^).

## SUPPLEMENTARY MATERIALS FIGURE AND TABLE





## Abbreviations

BC: breast cancer
ER: estrogen receptor
PR: progesterone receptor
EGFR: epidermal growth factor receptor
AREG: amphiregulin
EMT: epithelial to mesenchymal transition
KIBRA: kidney and brain expressed protein
YAP1: Yes-associated protein 1
TAZ: transcriptional co-activator with PDZ binding motif
MST1/2: mammalian Ste20-like serine/threonine kinase 1/2
LATS1/2: large tumor-suppressor kinase 1/2
SAV1: salvador family WW domain containing protein 1
PTPN14: non-receptor tyrosine phosphatase
β-TrCP: beta-transducin repeat containing E3 ubiquitin protein ligase
MTT: 3-(4,5-Dimethylthiazol-2-yl)-2,5-Diphenyltetrazolium Bromide
IGFBP6: insulin like growth factor binding protein 6
B-ALL: B-cell acute lymphocytic leukemia
CLL: chronic lymphocytic leukemia
GC: gastric cancer
ccRCC: clear cell renal cell carcinomas
CSC: cancer stem cell
TCGA: The Cancer Genome Atlas
OS: overall survival
RFS: relapse free survival
