# Supplementary material for: Loss of KIBRA function activates EGFR signaling by inducing AREG
Source: Oncotarget. 2018 Jul 6;9(52):29975–84. doi: 10.18632/oncotarget.25724 (PMC6057453; doi:10.18632/oncotarget.25724)
Supplement: Supplementary file 1 [file oncotarget-09-29975-s001.pdf]

Loss of KIBRA function activates EGFR signaling by inducing AREG

SUPPLEMENTARY MATERIALS

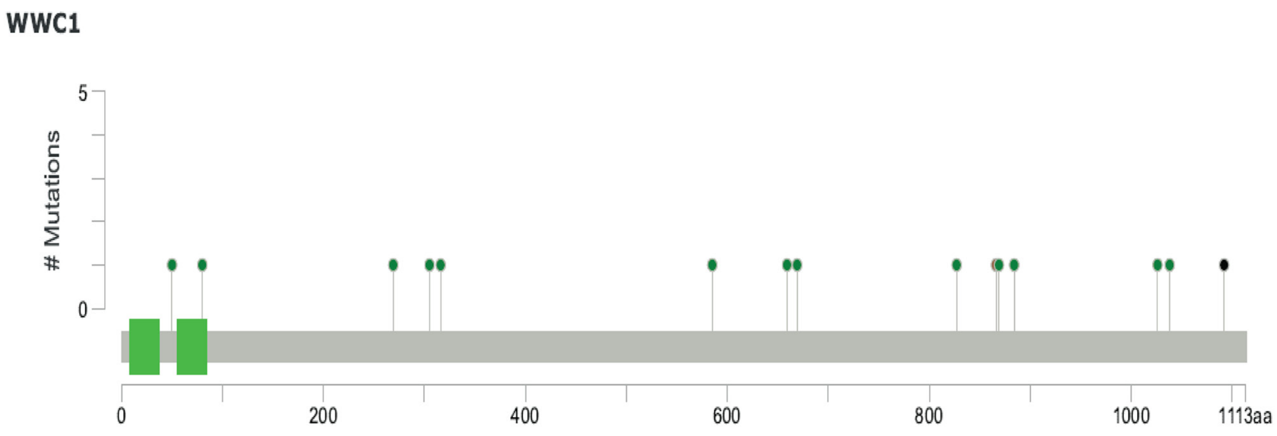

Supplementary Figure 1: KIBRA mutations in breast cancer patients were analyzed using TCGA data.

Supplementary Table 1: KIBRA mutations in breast cancer patients.

See Supplementary File 1
